# A rare Lisfranc-type injury involving dorsal dislocation of the intermediate cuneiform and second metatarsal

**DOI:** 10.1016/j.radcr.2024.10.083

**Published:** 2024-11-06

**Authors:** Morteza Gholipour, Mohsen Salimi, Alireza Motamedi, Fatemeh Abbasi

**Affiliations:** a Clinical Research Development Unit of Akhtar Hospital, Shahid Beheshti University of Medical Science, Tehran, Iran; b Student Research Committee, School of Medicine, Shiraz University of Medical Sciences, Shiraz, Iran; c Student Research Committee, Faculty of Medicine, Tabriz University of Medical Sciences, Tabriz, Iran; d Student Research Committee, Faculty of Medicine, Mazandaran University of Medical Sciences, Mazandaran, Iran

**Keywords:** Lisfranc injury, Dislocation, Tarsometatarsal joints, Fracture

## Abstract

Lisfranc injuries, involving the tarsometatarsal joints, are rare and account for approximately 0.2% of all fractures. Among these, dorsal dislocation of the intermediate cuneiform is extremely uncommon. This case study presents a 25-year-old male with a rare Lisfranc injury involving dorsal dislocation of the intermediate cuneiform and second metatarsal, following a motorcycle accident. Diagnosis was confirmed through radiographs and CT scans. The patient underwent closed reduction with percutaneous pinning and K-wire fixation. Postoperative outcomes were favourable, with the patient achieving full recovery and no residual pain. This report highlights the importance of accurate diagnosis and timely intervention to prevent long-term complications. The discussion includes a review of the Lisfranc joint anatomy, classification of injuries, and imaging techniques essential for proper evaluation. The case underscores the need for heightened clinical awareness and systematic imaging approaches in managing such rare injuries.

## Introduction

The Lisfranc joint complex consists of the tarsometatarsal joints, which include the cuneiforms, cuboids, and their articulations with the 5 metatarsal bases. this joint complex forms the foundation of the foot's longitudinal and transverse arches [1]. A Lisfranc injury involves disruption of this complex which includes fractures, dislocation, or displacement of one or more metatarsals [[1], [2], [3]].

Lisfranc injuries are rare and account for approximately 0.2% of all fractures. Among these, dorsal dislocation of the intermediate cuneiform is extremely uncommon and has been reported in only a few case studies [1,4,5].

This type of injury is often difficult to diagnose and despite its low occurrence rate it is clinically significant due to its potential for long-term complications when treatment is inadequate or postponed because of an initial missed diagnosis [6,7].

Imaging is crucial for diagnosing Lisfranc fracture-dislocations. Since radiographic signs can be subtle and patients in many cases present with multiple traumas, employing a systematic approach and incorporating additional imaging modalities are important for proper evaluation of these injuries [6,8,9]. We present a rare case of a Lisfranc injury involving dorsal dislocation of the intermediate cuneiform. By reviewing its imaging features and providing a comprehensive discussion, we aim to address the current gap in the literature and assist clinicians in future encounters with this type of injury.

## Case presentation

A 25-year-old male was brought to the emergency department after being involved in a motorcycle accident. The patient had trauma and pain localized to his right lower extremity. Physical examination revealed bruising, swelling, and tenderness over the right foot with an open wound. He was unable to bear weight on the affected limb. Patient was hemodynamically stable and laboratory tests were all within normal limits. Vascular assessment showed no abnormalities, and sensory function was intact. There were no other injuries or complaints reported.

**Fig. 1:**
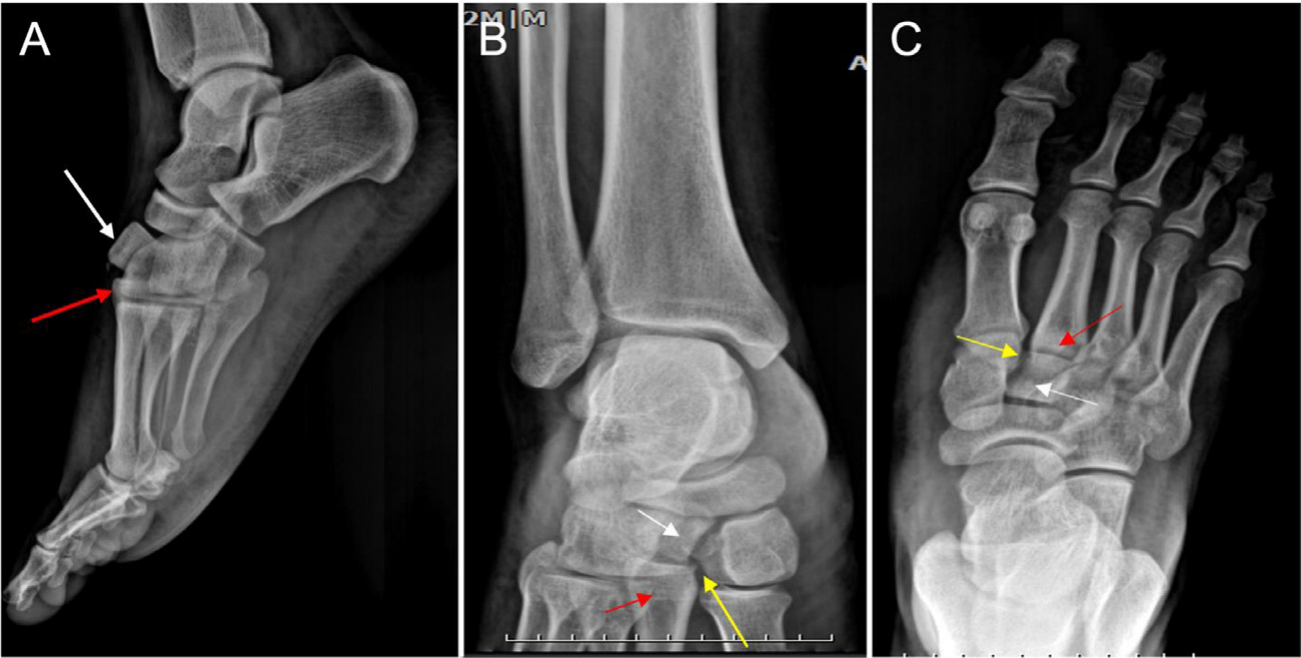
Plain radiographs of the right foot taken prior to surgery. (A) Lateral (mediolateral) view, (B) Oblique view, and (C) Anteroposterior (AP) view. The images clearly show a Lisfranc-type injury with dorsal dislocation of the intermediate cuneiform (white arrows) and second metatarsal (red arrows). Disruption is also seen between the medial cuneiform and the second metatarsal base, known as the “fleck sign” (yellow arrows).

Plain radiographs of the right foot revealed a dorsal dislocation of the intermediate cuneiform and the second metatarsal without any fractures detected (Fig. 1).

**Fig. 2:**
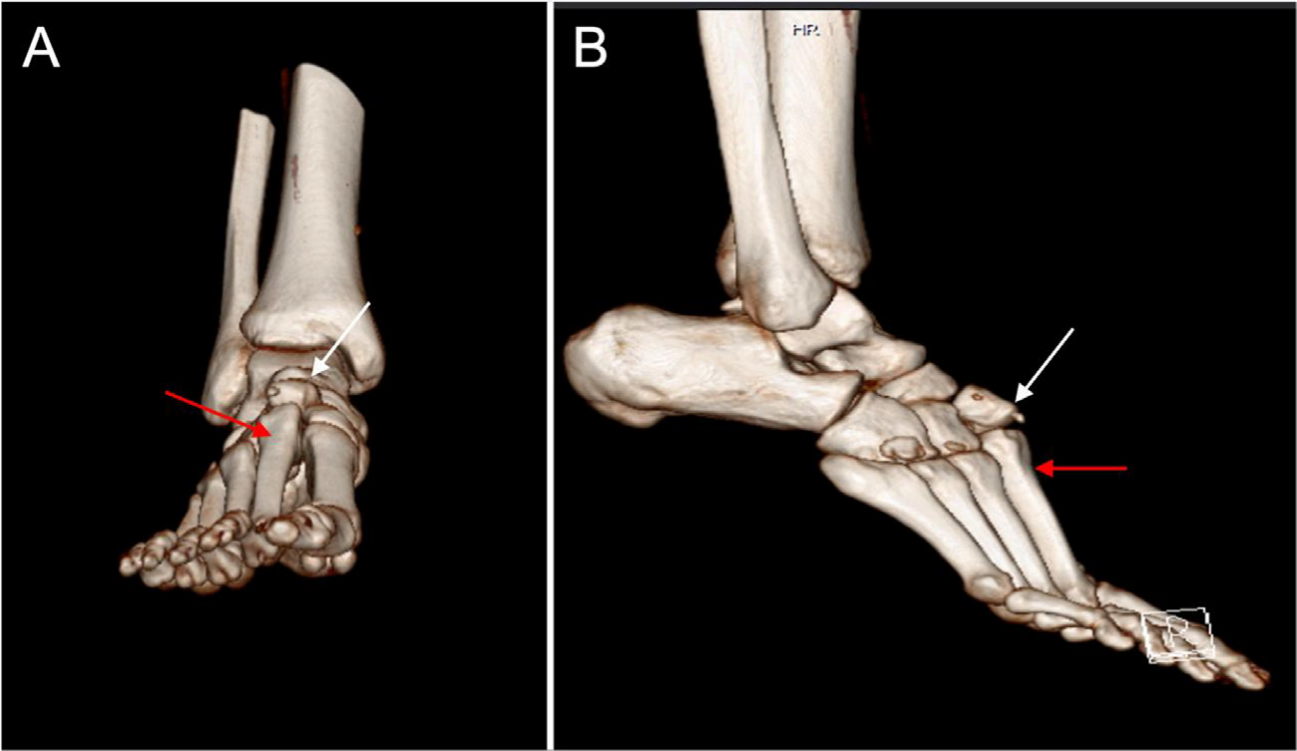
3D CT reconstruction images of the right foot prior to surgery. (A) Anterior view, and (B) Lateral view, showing a Lisfranc-type injury with dorsal dislocation of the intermediate cuneiform (white arrows) and second metatarsal (red arrows).

Computed tomography (CT) scans and 3-dimensional (3D) reconstructions of the right foot were performed, which supported the findings of radiographs (Fig. 2).

**Fig. 3:**
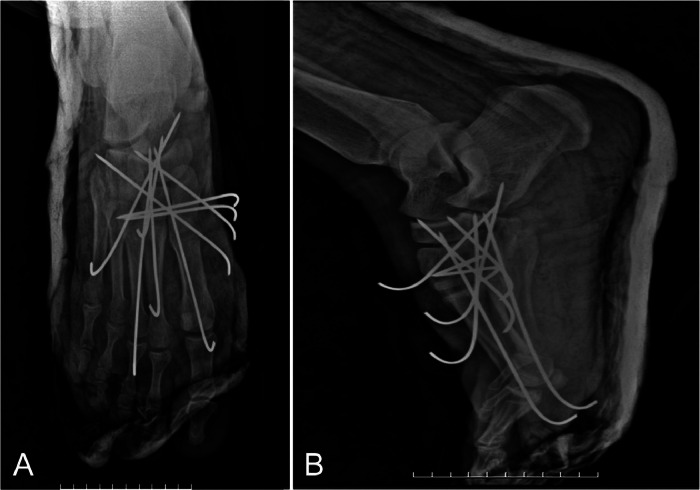
Plain radiographs of the right foot taken 1 day after surgery. (A) Anteroposterior (AP) view, (B) Lateral view. K-wire fixation and closed reduction were performed, and the medial cuneiform and metatarsals are in normal alignment.

The patient was taken to the operating room under fluoroscopic guidance using a C-arm, where a closed reduction with percutaneous pinning of the right midfoot was performed, and K-wires were used for internal fixation to stabilize the intermediate cuneiform and second metatarsal in oblique, lateral, and vertical planes. The Lisfranc injury was stabilized, and the foot was placed in a short leg splint in a nonweight-bearing position. The postoperative radiographs showed favorable results (Fig. 3).

**Fig. 4:**
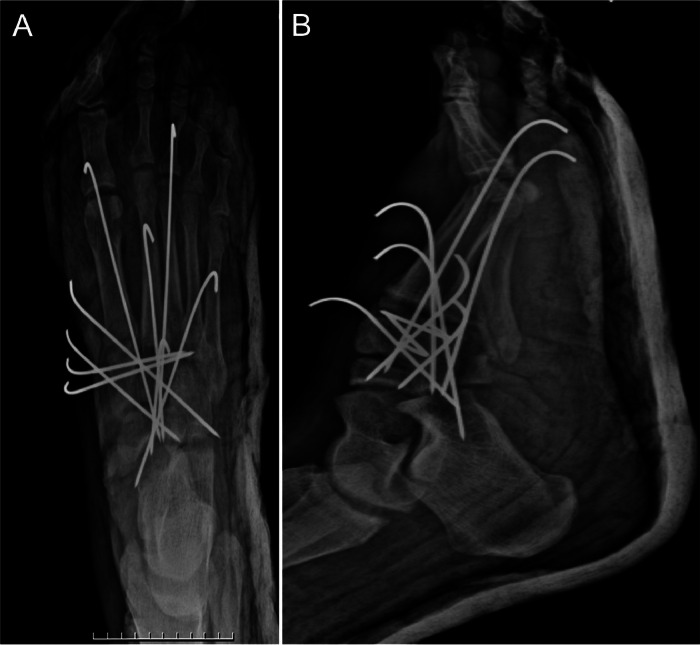
Follow-up plain radiographs of the right foot taken 2 weeks postoperation. (A) Anteroposterior (AP) view, (B) Lateral view. The medial cuneiform and metatarsals remain in normal alignment.

**Fig. 5:**
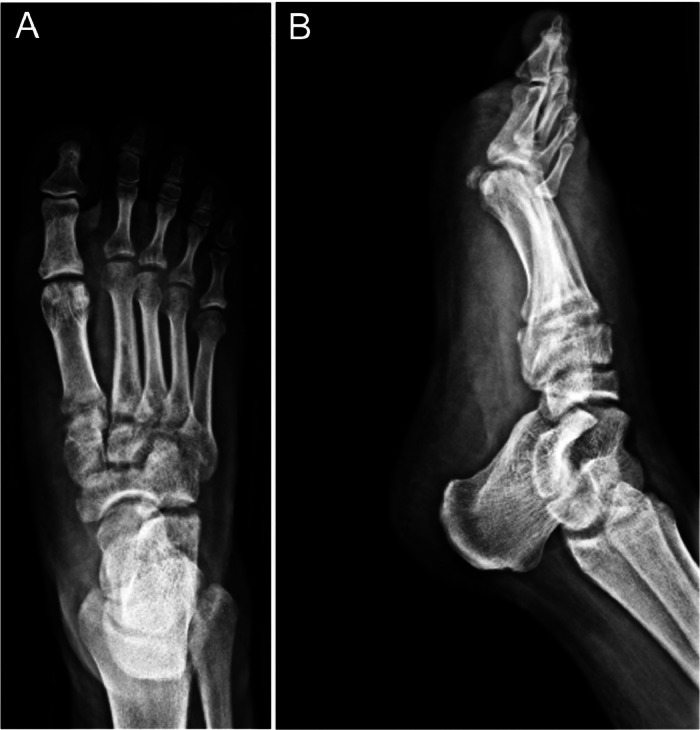
Follow-up plain radiographs of the right foot taken 6 weeks postoperation. (A) Anteroposterior (AP) view, (B) Lateral view. Pins were removed 6 weeks after surgery, and weight-bearing activities were initiated. The medial cuneiform and metatarsals remain in normal alignment, with no abnormalities observed.

Control and follow-up plain radiographs taken at 2 and 6 weeks showed positive outcomes (Figs. 4 and 5).

## Patient outcome

The pins were removed 6 weeks after surgery and weight-bearing activities were initiated 1 month later. At the final follow-up, the patient reported no foot pain with a full range of motion along with normal vascular and sensory examination findings.

## Discussion

The foot's anatomy is organized into 3 longitudinal columns that provide stability to the Lisfranc joint, also known as the tarsometatarsal (TMT) joint. The medial column consists of the medial cuneiform, navicular, and first metatarsal. The middle column includes the intermediate and lateral cuneiforms with the second and third metatarsals. The lateral column is made up of the cuboid with the fourth and fifth metatarsals [10,11]. These columns form the longitudinal and transverse arches of the foot, which are crucial for maintaining the joint's stability. The Lisfranc joint is further supported by strong ligamentous attachments, such as the intermetatarsal and tarsometatarsal ligaments, which have a strong plantar component and a relatively weaker dorsal component [6,[10], [11], [12]].

The classification of Lisfranc injuries was first established by Quenu and Kuss in 1909, based on the 3-column concept. This classification identifies the injuries into three categories: homolateral, isolated, and divergent [13]. The classification was later revised by Hardcastle in 1982 into 3 categories: A, B, and C, which remain widely used in clinical practice today [14]. Myerson et al. proposed a further modified classification system to account for the complexity of Lisfranc injuries [15].

The modified classification of Lisfranc injuries categorizes them into Type A, involving total incongruity with all metatarsals displaced in 1 direction; Type B, with partial incongruity subdivided into B1 (isolated medial dislocation of the first metatarsal) and B2 (isolated lateral dislocation of the second to fifth metatarsals); and Type C, showing a divergent pattern, further divided into C1 (divergent injury of some tarsometatarsal joints) and C2 (divergent injury of all tarsometatarsal joints) [15].

Lisfranc injuries may occur due to direct or indirect trauma with direct trauma typically seen in motorcycle accidents like our case and indirect trauma more commonly found in athletes [2,16,17].

Clinical presentation typically includes foot deformity with severe pain, swelling, and an inability to bear weight. Open injuries with damage to the skin and underlying tissues may also be present. In polytrauma patients, these injuries should not be missed due to the risk of compartment syndrome and neurovascular compromise, both of which require immediate treatment [[18], [19], [20]].

Lisfranc injuries can be first discovered through nonweight-bearing (NWB) plain radiographs, however this approach may not be able to detect many of the more subtle injuries [21,22].

Initially, anteroposterior (AP), oblique, and lateral radiographs of the foot should be obtained to assess key anatomical alignments. On the AP view, the medial side of the middle cuneiform should align with the medial side of the second metatarsal. The 30-degree oblique view should show the medial side of the lateral cuneiform in line with the base of the third metatarsal and the medial edge of the cuboid. In the lateral view, the metatarsals should not appear displaced above or below their corresponding tarsal bones. Any misalignment in these relationships indicates a possible Lisfranc joint injury [10].

The most frequent radiographic indicator of a Lisfranc joint injury is a separation (diastasis) between the bases of the first and second metatarsals. Additionally, a gap greater than 2 mm between the bases of the first and second metatarsals warrants further assessment, including comparison with the uninjured foot [23].

Another sign on radiographs is the “fleck sign,” which appears as a small bone fragment located between the bases of the first and second metatarsals, in the Lisfranc space. This fragment typically results from a tearing away at the attachment site of the Lisfranc ligament, either at the base of the second metatarsal or the medial cuneiform. Though it may be subtle, the fleck sign is a critical indication of a possible Lisfranc injury [1,24,25].

Weight-bearing radiographic views can be useful in cases where a Lisfranc injury is suspected, but nonweight-bearing (NWB) radiographs do not reveal any abnormalities [19].

Minor displacements may only be detectable through a CT scan or Magnetic Resonance Imaging (MRI). These imaging modalities are recommended as complementary tools, especially in cases involving multiple trauma [1,26,27].

The main aim of treatment is to reconstruct the midfoot's anatomy to maintain its functionality and reduce the risk of developing arthritis and disability later on [28].

Nonoperative treatment is recommended for Stage 1 stable Lisfranc injuries, which have a minimal <2 mm separation (diastasis) between the first and second metatarsals and no associated fractures [1,19,22].

Surgical intervention is recommended for Lisfranc injuries when there is a diastasis greater than 2 mm between the bases of the first and second metatarsals or injuries with displacement [7,23,29].

Surgical interventions for Lisfranc injuries can differ, but they typically involve using screws to stabilize the Lisfranc ligament by securing the medial cuneiform to the second metatarsal. Additional stabilization is commonly achieved by fixing the first metatarsal to the medial cuneiform and the second metatarsal to the middle cuneiform [10,30].

An alternate approach involves performing a closed reduction and using K-wire fixation to achieve definitive stabilization. However, the available evidence suggests that screw fixation provides enhanced stability for the medial and intermediate columns [[30], [31], [32]].

In our case, K-wire fixation and closed reduction were chosen based on the surgeon's personal preference, considering the patient's condition.

## Conclusion

In conclusion, Lisfranc injuries, including the extremely rare dorsal dislocation of the intermediate cuneiform, require precise diagnosis and tailored management to prevent long-term complications. Treatment approaches vary based on the degree of displacement and stability, highlighting the importance of appropriate imaging and timely intervention to achieve the best outcomes.

## Patient consent

Written informed consent was obtained from the patient for publication and any accompanying images. A copy of the written consent is available for review by the Editor-in-Chief of this journal on request.
